# Enhanced direct fermentation of cassava to butanol by *Clostridium* species strain BOH3 in cofactor-mediated medium

**DOI:** 10.1186/s13068-015-0351-7

**Published:** 2015-10-12

**Authors:** Tinggang Li, Yu Yan, Jianzhong He

**Affiliations:** Department of Civil and Environmental Engineering, National University of Singapore, Block E2-02-13, 1 Engineering Drive 3, Singapore, 117576 Singapore

**Keywords:** Butanol, *Clostridium* sp., Cofactor, α-amylase, Simultaneous saccharification and fermentation, Cassava

## Abstract

**Background:**

The main challenge of cassava-based biobutanol production is to enhance the simultaneous saccharification and fermentation with high hyperamylolytic activity and butanol yield. Manipulation of cofactor [e.g., Ca^2+^ and NAD/(P)H] levels as a potential tool to modulate carbon flux plays a key role in the cassava hydrolysis capacity and butanol productivity. Here, we aimed to develop a technology for enhancing butanol production with simultaneous hydrolysis of cassava (a typical model as a non-cereal starchy material) using a cofactor-dependent modulation method to maximize the production efficacy of biobutanol by *Clostridium* sp. stain BOH3.

**Results:**

Supplementing CaCO_3_ to the medium containing cassava significantly promotes activities of α-amylase responsible for cassava hydrolysis and butanol production due to the role of Ca^2+^ cofactor-dependent pathway in conversion of cassava starch to reducing sugar and its buffering capacity. Also, after applying redox modulation with l-tryptophan (a precursor as de novo synthesis of NADH and NADPH), the levels of cofactor NADH and NADPH increased significantly by 67 % in the native cofactor-dependent system of the wild-type *Clostridium* sp. stain BOH3. Increasing availability of NADH and NADPH improved activities of NADH- and NADPH-dependent butanol dehydrogenases, and thus could selectively open the valve of carbon flux toward the more reduced product, butanol, against the more oxidized acid or acetone products. By combining CaCO_3_ and l-tryptophan, 17.8 g/L butanol with a yield of 30 % and a productivity of 0.25 g/L h was obtained with a hydrolytic capacity of 88 % towards cassava in a defined medium. The metabolic patterns were shifted towards more reduced metabolites as reflected by higher butanol–acetone ratio (76 %) and butanol–bioacid ratio (500 %).

**Conclusions:**

The strategy of altering enzyme cofactor supply may provide an alternative tool to enhance the stimulation of saccharification and fermentation in a cofactor-dependent production system. While genetic engineering focuses on strain improvement to enhance butanol production, cofactor technology can fully exploit the productivity of a strain and maximize the production efficiency.

**Electronic supplementary material:**

The online version of this article (doi:10.1186/s13068-015-0351-7) contains supplementary material, which is available to authorized users.

## Background

Renewable biofuel is one of the options to solve potential energy crisis, limited supply of petroleum fuels, environmental and climate change problems [1, 2]. Biobutanol produced naturally by solventogenic *Clostridium* species is considered to be a new generation of biofuel in contrast to bioethanol (as the current major biofuel), and thus has the potential to replace gasoline or as a fuel additive [3]. The industrial-scale production of butanol through fermentation has recently urged to use renewable biomass [4]. However, over the years, the major limitations associated with acetone–butanol–ethanol (ABE) fermentation include product toxicity to the solventogenic bacteria (i.e., butanol tolerance), substrate to product conversion efficiency, the ability to utilize inexpensive biomass as a substrate (i.e., substrate cost), and the potential for culture degeneration, which made the industrial-scale fermentation-derived butanol less competitive when compared to the petroleum-based butanol production [1, 5].

To improve the economic competitiveness of biobutanol production, from fermentation substrate point of view, the availability of an inexpensive raw material is essential as the cost of the substrate is one of the most important factors affecting the market price of butanol [6, 7]. Cassava, a non-cereal starchy crop, has been widely grown in poor soils and harsh climates than any other major food plant in Southeast Asia. The world cassava output in 2010 was 242 million tonnes, and reached 282 million tonnes in 2012 [8]. The production of cassava in Southeast Asia accounted for ~25.4 % of the current world production of cassava. Thus, cassava represents an alternative cheap carbon source for industrial production of butanol, which is promising and attractive in both economic and geographical considerations.

Using conventional cassava-based ABE fermentation, an additional pretreatment process is obligatorily required for its two steps with saccharification by acids or enzymes to enhance starch hydrolysis, following by microbial fermentation [9]. However, the production of amylolytic enzymes by a single strain that also metabolizes the sugars produced to the desired end product has an advantage in terms of cost over those systems where hydrolysis and subsequent fermentation of starch occur separately [10, 11]. Therefore, the main challenge of cassava-based biobutanol production is how to enhance the simultaneous saccharification and fermentation (SSF) with high hyperamylolytic activity and butanol yield. The saccharolytic solventogenic *Clostridium* include 5 main enzymes, among which α-amylase is one of the most important amylolytic enzymes [10], because the level and activity of α-amylase determine the rate of starch hydrolysis—a rate-limiting step in conversion of starch into reducing sugars for following usage by solventogenic *Clostridium* sp. [12, 13]. Since the rate of hydrolysis of cassava starch by α-amylase depends on many factors such as nature of substrate and presence of cofactors such as calcium ion (Ca^2+^) [14–16], there is a need to investigate how Ca^2+^ cofactor influences α-amylase activity which is responsible for conversion of cassava starch to fermentable reducing sugars for following butanol fermentation.

The level and availability of reducing cofactor is another critical parameter in cofactor-dependent biobutanol production systems, especially the solventogenic *Clostridium* sp. involving NADH- and NADPH-dependent butanol dehydrogenases [17]. Consequently, a lack or inefficient regeneration of the cofactor NADH or NADPH would shutdown the butanol dehydrogenase activity. Cofactor manipulations can potentially become a powerful tool for improvement of overall process yield and productivity [18, 19]. Therefore, a feasible way to improve the economic efficacy of butanol production is to maximize cassava hydrolysis and the butanol yield in the cassava-based direct ABE fermentation. Our hypotheses are that (1) the extracellular α-amylase of *Clostridium* sp. strain BOH3 requires Ca^2+^ as a metal cofactor for efficient hydrolysis of cassava starch into reducing sugars; (2) a lack or inefficient regenerations of the cofactor NADH or NADPH would shutdown the butanol dehydrogenase reaction, resulting in a low butanol titer. Thus, we first evaluated changes in enzymatic and mRNA levels of α-amylase in *Clostridium* sp. strain BOH3 without and with exogenous CaCO_3_. Second, we supplemented l-tryptophan, a precursor in de novo synthesis of NADH and NADPH, in the defined medium to enhance the reducing force to redirect the flux via triggering the availabilities of NADH and NADPH. The above studies highlight the two key cofactors for efficient ABE fermentation from cassava.

## Results

### Calcium as a metal cofactor in the α-amylase of *Clostridium* sp. strain BOH3

**Fig. 1:**
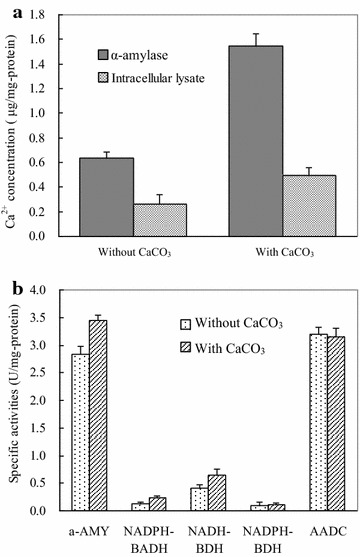
Calcium as a metal cofactor in the α-amylase of *Clostridium* sp. strain BOH3. **a** Ca^2+^ concentration in α-amylase and cell lysate without or with supplementation of CaCO_3_; **b** CaCO_3_-induced increases in the activities of α-amylase, NADPH- and NADH-dependent butyraldehyde and butanol dehydrogenases. Increases in activity are expressed as fold differences between the CaCO_3_-supplemented fermentation broth and the CaCO_3_-free fermentation broth

Some α-amylases are considered to be metallic hydrolases which require calcium ion (Ca^2+^) for their activity, structural integrity and stability [20, 21], while other α-amylases are reported to be Ca^2+^-independent in microbial reaction [15, 16]. Through using a Ca^2+^ probe fluorescence assay, we investigated if calcium exists in the α-amylase as a metal cofactor and there are increases in intracellular Ca^2+^ levels in *Clostridium* sp. strain BOH3 when it is growing in Ca^2+^-supplemented medium compared to the control medium. Ca^2+^ was detected in pure α-amylase secreted by culture BOH3 without Ca^2+^ treatment, suggesting Ca^2+^ is a metal cofactor for extracellular α-amylase of culture BOH3 (Fig. 1a). Furthermore, α-amylase secreted from Ca^2+^-supplemented culture medium and BOH3 cells showed 143.1 and 89.7 % higher intracellular Ca^2+^ concentration than those in the Ca^2+^-free culture medium at the late exponential fermentation phase at 36 h (1.54 vs 0.63 and 0.49 vs 0.26 μg Ca^2+^/mg-protein). On the other hand, the activity of α-amylase increased 21.9 % when 10 mM Ca^2+^ was introduced into the cassava-defined medium (Fig. 1b). The higher intracellular Ca^2+^ concentration and enhanced α-amylase activity in the Ca^2+^-supplemented culture medium strongly suggest that Ca^2+^ serves as a metal cofactor and plays specific physiological/cellular roles. Therefore, following studies was conducted to understand whether the increase of Ca^2+^ (as a CaCO_3_) in fermentation medium could pose stimulatory effects on cassava starch hydrolysis and solventogenesis, and then enhance the butanol production.

### Effects of CaCO_3_ on direct butanol production from cassava by strain BOH3

To improve hydrolytic capacity of cassava flour and solvent production, the medium was initially supplemented with a mixture of ingredients (e.g., yeast extract, and hydrolases). However, the cost of these supplemental ingredients would be prohibitive in industrial-scale applications. Interestingly, a series of screening tests on the ingredient(s) (e.g., alanine, cysteine, glutathione, CuCl_2_, ZnCl_2_, and CaCO_3_) for increased ABE production identified calcium carbonate as an effective ingredient in the supplement mixture. Therefore, to determine the dose-dependent effects of CaCO_3_ on starch hydrolysis and solvent production by strain BOH3, the medium was amended with 0, 5, 10, 30, 50, 70 or 100 mM of CaCO_3_. As shown in Table 1, the amount of cassava starch consumption and butanol productivity along with more than 10 mM CaCO_3_ supplement increased by 78.6 and 56.3 %, as compared with that of the control (absence of CaCO_3_). Second, the amount of biomass (DCW) was 44.1 % higher than that without CaCO_3_ addition. Third, the highest concentrations of butanol and total solvent (11.8 ± 0.24 and 16.0 ± 1.1 g/L) were achieved in the presence of 10 mM CaCO_3_. Fourth, the lowest pH of the culture (3.9 ± 0.2) was observed in the absence of CaCO_3_, lower than that in the presence of CaCO_3_, while final pH of fermentation broth in the presence of CaCO_3_ (100 mM) was 17 % higher than that of control without CaCO_3_. Lastly, the higher level (>10 mM) of CaCO_3_ did not affect the cell growth and butanol production, meanwhile acting as a buffer reagent for fermentation culture. However, acetone, the main by-product of ABE fermentation, decreased slightly when CaCO_3_ was more than 10 mM in the culture medium (Table 1).

**Table 1:** Effect of calcium carbonate on ABE production by wild-type *Clostridium* sp. strain BOH3

Fermentation characteristics^a^	Calcium carbonate (mM)						
	0	5	10	30	50	70	100
Maximum dry cell weight (g/L)	3.4 ± 0.18	4.0 ± 0.23	4.9 ± 0.19	4.8 ± 0.20	4.7 ± 0.24	4.7 ± 0.21	4.5 ± 0.22
Butanol (g/L)	7.8 ± 0.13	9.5 ± 0.31	11.8 ± 0.24	11.6 ± 0.41	11.8 ± 0.32	11.4 ± 0.29	11.0 ± 0.35
Acetone (g/L)	3.8 ± 0.17	3.6 ± 0.25	3.3 ± 0.21	3.1 ± 0.21	3.0 ± 0.17	2.8 ± 0.15	2.6 ± 0.19
Ethanol (g/L)	0.7 ± 0.01	0.8 ± 0.02	0.9 ± 0.06	0.8 ± 0.05	0.7 ± 0.04	0.6 ± 0.02	0.6 ± 0.04
Acetate (g/L)	2.1 ± 0.07	3.8 ± 0.19	1.7 ± 0.04	1.5 ± 0.08	1.4 ± 0.06	1.1 ± 0.09	0.8 ± 0.07
Butyrate (g/L)	2.4 ± 0.11	3.4 ± 0.16	1.1 ± 0.01	0.7 ± 0.05	0.6 ± 0.07	0.8 ± 0.02	0.5 ± 0.03
Total solvent (g/L)	12.3 ± 0.5	13.9 ± 0.9	16.0 ± 1.1	15.5 ± 1.2	15.5 ± 0.8	14.8 ± 1.0	14.2 ± 0.9
pH_lowest_^a^	3.9 ± 0.2	4.1 ± 0.1	4.8 ± 0.2	4.9 ± 0.3	5.1 ± 0.3	5.2 ± 0.2	5.3 ± 0.1
Final pH	4.7 ± 0.1	4.8 ± 0.2	5.0 ± 0.1	5.2 ± 0.2	5.3 ± 0.1	5.4 ± 0.2	5.5 ± 0.2
Butanol productivity (g/L h)	0.10 ± 0.01	0.13 ± 0.01	0.16 ± 0.01	0.16 ± 0.01	0.16 ± 0.01	0.16 ± 0.02	0.15 ± 0.02
Total starch hydrolysis rate (g/L h)	0.48 ± 0.02	0.59 ± 0.03	0.75 ± 0.02	0.73 ± 0.04	0.75 ± 0.03	0.75 ± 0.01	0.73 ± 0.04

^a^No pH adjustments

**Fig. 2:**
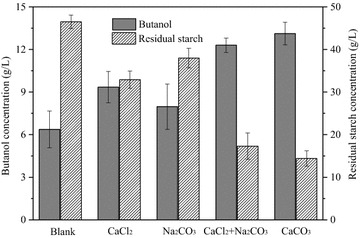
Role of calcium and carbonate ions on butanol production by strain BOH3 in defined medium amended with cassava. *Blank* stands for absence of CaCO_3_, CaCl_2_ stands for 10 mM CaCl_2_, Na_2_CO_3_ stands for 10 mM Na_2_CO_3_, CaCl_2_ + Na_2_CO_3_ stands for 10 mM CaCl_2_ and 10 mM Na_2_CO_3_, CaCO_3_ stands for 10 mM CaCO_3_. The fermentation was performed at 37 °C for 96 h. Initial cassava flour concentration is 100 g/L containing 68 g/L of starch. Data represent the mean ± SD of triplicate individual experiments

Having demonstrated that CaCO_3_ enhanced cassava saccharification and following fermentation by culture BOH3, further experiments were conducted to understand whether it was the calcium (Ca^2+^) or carbonate (CO_3_^2−^) taking effects on cassava starch hydrolysis and butanol production. *Clostridium* sp. strain BOH3 was grown on a cassava amended medium supplemented with either a calcium source—calcium chloride (CaCl_2_) or a carbonate source—sodium carbonate (Na_2_CO_3_). Although no pH adjustments, increases of butanol production and cassava starch consumption proved the favorable effect of carbonate on ABE fermentation as conferred by the partial buffering capacities as compared to the control (Fig. 2). Furthermore, the addition of CaCl_2_ to the medium clearly demonstrated that the effects of CaCO_3_ did not stem solely from its buffering effect. While both Ca^2+^ (10 mM) and CO_3_^2−^ (10 mM) ions were included in cassava medium, butanol production and cassava starch consumption were enhanced more than 35.2 and 33.8 % when comparing with sole CO_3_^2−^ ion (10 Mm) addition, 31.5 and 23.9 % when comparing with sole Ca^2+^ ion (10 mM) addition. The results were comparable with that achieved by adding 10 mM CaCO_3_ (Fig. 2). We therefore conclude that both Ca^2+^ and CO_3_^2−^ ions were required for improving cassava hydrolysis rate and enhancing solvent production by strain BOH3.

### Further confirmation of the role of CaCO_3_ from enzymatic studies

**Fig. 3:**
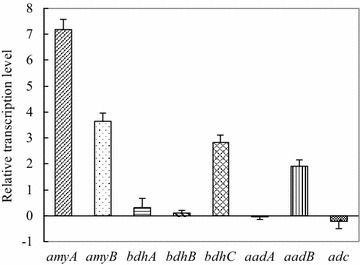
Real-time PCR analysis of gene transcripts involved in hydrolysis and solventogenesis. Changes were illustrated for selected genes of *Clostridium* sp. strain BOH3 at 36 h of fermentation (at the end-exponential phase) with or without CaCO_3_. Relative expression levels confirmed the upregulation of *amyA* and *amyB* encoding α-amylase (α-AMY), *bdhA, bdhB*, and *bdhC* encoding butanol dehydrogenase (BDH), and down-regulation of *aadB* encoding alcohol/aldehyde dehydrogenase (AAD), and *adc* encoding acetoacetate decarboxylase (AADC)

To independently confirm the role of calcium carbonate on modulated starch hydrolysis and solvent generation at the molecular level, transcription analysis of functional genes in *Clostridium* sp. strain BOH3 was conducted by real-time PCR using cells growing in cassava medium amended with and without calcium carbonate (Fig. 3). Transcriptions of amylolytic (*α*-*amyA and α*-*amyB*) and key solventogenic genes (*bdhA*, *bdhC* and *aadB*) in strain BOH3 increased in the cassava medium supplemented with calcium carbonate comparing with calcium carbonate-free medium, while transcriptions of solventogenic *adc and aadA* genes in strain BOH3 decreased. The transcriptional upregulation of *α*-*amyA* and *α*-*amyB* was consistent with higher cassava degradation rate during starch hydrolysis phase. It was noted that levels of *adc* and *aadA* mRNAs were down-regulated by 2.4 and 11.5 % in the CaCO_3_-supplemented cultures relative to the controls (Fig. 3), corroborating negative changes in their respective enzyme activities in our key enzyme studies (Fig. 1b). This suggested that these two genes may not be divalent ion-dependent genes.

Subsequently, to verify whether CaCO_3_ modulates the activities of important enzymes involved in ABE production from cassava, aliquots of supernatant and cell extracts of strain BOH3 in the presence or absence of 10 mM CaCO_3_ were assayed for α-amylase (α-AMY), butyraldehyde dehydrogenase (BADH), and butanol dehydrogenase (BDH), alcohol/aldehyde dehydrogenase (AAD), and acetoacetate decarboxylase (AADC) activities (Fig. 1b). An increase of 21.9, 76.9, and 58.8 % activity was observed for α-AMY, NADH-dependent BADH, and NADH-dependent BDH, respectively, when 10 mM Ca^2+^ was introduced in the medium. BADH and BDH are NADH-dependent quinohaemoprotein, while Ca^2+^ ion facilitated the reconstitution of inactive apoenzyme (pyrroloquinoline quinone) quinohaemoprotein so as to increase the BADH and BDH catalytic activity [22, 23]. A negligible difference in activity was observed for NADPH-dependent BDH upon Ca^2+^ supplementation in fermentation medium. However, a dramatic decrease in AADC activity was observed in the presence of CaCO_3_, which was responsible for acetone production—the main by-product during fermentation.

### Tryptophan-induced redox modulation of ABE fermentation by strain BOH3

**Fig. 4:**
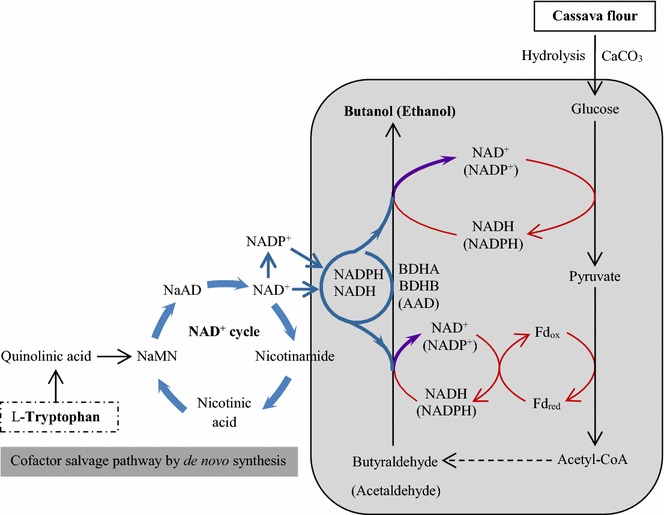
Relationship of alcohol synthesis and NADH/NADPH regenerations. The de novo biosynthesis of NADH/NADPH starts at l-tryptophan and then NADH/NADPH goes to butanol pathway via NADPH/NADH circle while NAD^+^/NADP^+^ are generated; beside complement of NADH/NADPH from de novo synthesis, NAD^+^/NADP^+^ in active cells goes back to NADPH/NADH to regenerate its reducing form. nicotinic acid mononucleotide (NaMN); nicotinic acid adenine dinucleotide (NaAD); nicotinamide adenine dinucleotide (NAD^+^); nicotinamide adenine dinucleotide hydrogen (NADH); nicotinamide adenine dinucleotide phosphate (NADP^+^); nicotinamide adenine dinucleotide phosphate hydrogen (NADPH); alcohol/aldehyde dehydrogenase (AAD); butanol dehydrogenase I (BDHA); butanol dehydrogenase II (BDHB)

**Fig. 5:**
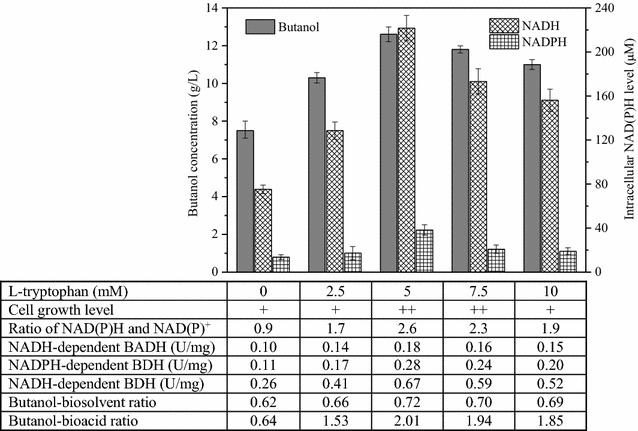
Comparison of intracellular NADH and NADPH levels and butanol production by altering l-tryptophan level. The extent of anaerobic growth is indicated where one “+” refers to an OD_600_ of around 1.6–2.0 in cultures. Changes in activities were observed for butyraldehyde dehydrogenase (BADH) or butanol dehydrogenase (BDH) which requires redox cofactors (NADH and NADPH) during butanol synthesis. Redistributed carbon flux to butanol in terms of butnaol–biosolvent ratio and butanol–bioacid ratio was specified in the table at the *bottom* by manipulating l-tryptophan levels

Cofactor availability and the active proportion of cofactor may play an important role in determining the overall process yield in cofactor-dependent production systems, such as ABE fermentation by *Clostridium* sp. in which butanol biosynthesis requires reducing power from NAD/(P)H. To increase the intracellular level of the cofactor NAD/(P)H, l-tryptophan—a precursor of the cofactor NAD/(P)H in *de novo* synthetic pathway (see Fig. 4) was supplemented at various dosage into the defined medium amended with cassava. The total NADH and NADPH levels increased with the increase of l-tryptophan dosage and reached a maximum of 221.7 and 38.3 µM at 5 mM l-tryptophan supplement (Fig. 5). In contrast to the control (absence of l-tryptophan), the enhanced ratios from 0.9 to 2.6 [ratio of NAD/(P)H to NAD/(P)^+^] indicate that more reducing equivalents were generated in the forms of NADH and NADPH. Meanwhile, the corresponding redox cofactor-dependent enzymes, the NADH- and NADPH-dependent BDH, and NADH-dependent BADH activities were also observed to enhance by 80, 155 and 158 % when triggering cofactor de novo synthesis (supplement of precursor l-tryptophan) compared to the control (Fig. 5), indicating that sufficient NADH and NADPH are the key in improving *bdh* gene expressions. As expected, higher butanol concentration (~12.6 vs 7.5 g/L) was obtained with higher butanol–biosolvent ratio (0.72 vs 0.62) and butanol–bioacid ratio (2.01 vs 0.64) when 5 mM l-tryptophan was dosed to the medium (Fig. 5). Clearly, supplement of l-tryptophan increased NAD/(P)H availability and drove more metabolic flux toward the more reduced product, butanol, against the more oxidized acid products. These results suggest that the salvaging reducing cofactors did play an important and specific role in channeling the carbon flux into the synthetic NAD/(P)H-dependent butanol pathway.

### Direct fermentation of cassava to butanol by strain BOH3 in a bioreactor supplemented with a combination of CaCO_3_ and l-tryptophan

**Fig. 6:**
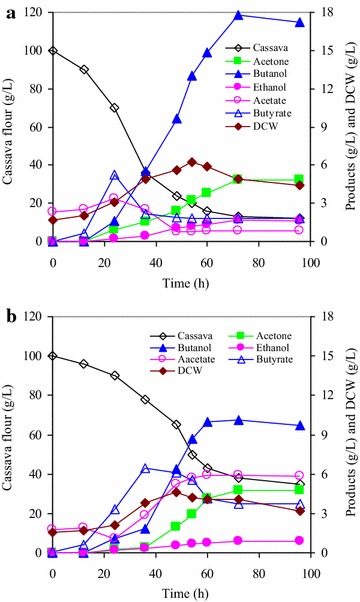
Direct conversion of cassava to butanol in a bioreactor by *Clostridium* sp. strain BOH3. **a** With supplement of CaCO_3_ and l-tryptophan; **b** without supplement of CaCO_3_ and l-tryptophan (control). Experiments were conducted in triplicate in terms of robustness of culture BOH3, metabolites and cassava utilization rate, although the results for one fermentation are shown. Differences in product formation between the triplicates were less than 5 %

To further strengthen cassava hydrolysis rate and the reducing force and to minimize the adverse effects of cultivation process (e.g., sharp pH fluctuation from 6.0 to 4.1, and CO_2_ biogas accumulation and oversaturation in culture), direct fermentation of cassava was conducted in a 3-L stirred-tank bioreactor spiked with both CaCO_3_ (10 mM) and l-tryptophan (5 mM). Hydrolysis of cassava was fully improved (~91 vs ~62 %), and acetic acid and butyric acid were observed to be lower but peaked 24 h earlier than the control (Fig. 6). Notably, 17.8 g/L butanol and 24.2 g/L total solvent were produced from 100 g/L cassava, which increased by 75.1 and 53.2 %, respectively, as compared to the control without either of them (Fig. 6). Moreover, the butanol–acetone ratio and butanol–bioacid ratio were enhanced from 3.7 to 6.6 and dropped from 2.1 to 1.1 as compared to the control. More importantly, when one glucose molecule is converted to the C4 compound butanol instead of the C3 compound acetone, the mass yield is expected to increase. Collectively, butanol yield on starch increased to approximately 30 % (24 % for control), indicating that the organic carbon of cassava is flowing to the more reducing product, alcohol (mainly butanol) by strain BOH3 in the medium amended with both CaCO_3_ and l-tryptophan.

## Discussion

This work demonstrates the importance of cofactors in improving substrate utilization and butanol yield in the native cofactor-dependent production system of a wild-type *Clostridium* sp. strain BOH3. With optimizing the Ca^2+^ metal cofactor (from CaCO_3_) coupled with NAD/(P)H reducing cofactors (via addition of the precursor l-tryptophan), strain BOH3 hydrolyzes and ferments substrate cassava directly to butanol, reaching a concentration of 17.8 g/L, a yield of 0.3 g/g and a productivity of 0.25 g/L h (Table 2), which is comparable to those levels produced by other *Clostridium* species employing pretreatment such as commercial enzyme hydrolysis and coculture fermentation or mutation [7, 9, 24, 25]. Results in this study support our hypothesis that (1) the extracellular α-amylase of *Clostridium* sp. strain BOH3 requires Ca^2+^ as a metal cofactor to eliminate the limiting step of cassava starch hydrolysis to reducing sugars under condition of high glucose demand in solventogenic *Clostridium* sp., and (2) a lack or inefficient regenerations of reducing cofactors NADH and NADPH impair activities of butanol dehydrogenases.

**Table 2:** Performance of different pretreatment method during cassava-based butanol fermentation

Organism	Carbon source	Pretreatment	Butanol (g/L)	Ethanol (g/L)	Acetone (g/L)	Butanol yield (g/g)	Butanol productivity (g/L h)	Solvent productivity (g/L h)	Reference
*C. saccharoperbutylacetonicum* N1-4	Cassava starch	Enzymatic hydrolysis (commercial enzymes)^a^	17.5	1.1	2.7	0.33	0.24	0.30	[9]
*Bacillus subtilis* WD 161 and *C. butylicum* TISTR 1032^b^	Cassava starch	Coculture	6.7	Approx. 0.9	Approx. 2.1	0.21	0.09	0.14	[7]
*C. butyricum* TISTR1032	Cassava pulp and cassava wastewater	Enzymatic hydrolysis (commercial enzymes)^c^	2.5	1.6	0.6	0.10	–	–	[26]
*C. beijerinckii* ATCC 55025 and *C. tyrobutyricum* 25755^d^	Cassava starch	Coculture	6.7	1.5	4.0	0.18	0.96	1.93	[24]
*C. acetobutylicum* PW12	Cassava flour	No pretreatment	12.1	1.9	4.9	0.27	0.13	0.21	[25]
Mutant strain ART18 of *C. acetobutylicum* PW12	Cassava flour	No pretreatment	16.3	2.4	5.8	0.31	0.19	0.28	[25]
*Clostridium.* sp. strain BOH3	Cassava flour	No pretreatment	17.8	1.6	4.8	0.30	0.25	0.34	This study

*Approx.* approximately

^a^Commercial enzymes: granular starch hydrolyzing enzymes (α-amylase and glucoamylase)

^b^ABE and acids (acetic acid and butyric acid) concentrations are 9.7 and 7.3 g/L with yeast extract/NH_4_NO_3_ ratio of 265/100 (mM/mM) in coculture system, respectively

^c^Commercial enzymes: Liquozyme^®^ SC DS (α-amylase), Spirizyme^®^ Fuel (glucoamylase), and Novozyme^®^ NS 50012 (multienzyme complex)

^d^Immobilized coculture in two fibrous-bed bioreactors

Finding low-cost substrate or optimizing fermentation conditions are two key means to reduce the costs for biobutanol production. Cassava becomes one of the choices due to its readily available non-food substrates. However, starch utilization is often inefficient due to the low activity of the amylases produced by *Clostridium* species [7, 26]. This study found that Ca^2+^ stimulated hydrolysis capacity of cassava and inhibited by-product acetone production during saccharification and fermentation processes by culture BOH3. Most importantly, the fermentation broth additive calcium carbonate serves to increase the buffer capacity (relieving accumulation of acetic acid) and butanol tolerance and productivity [27, 28]. Evidence of uptake of Ca^2+^ suggests that amylolytic α-amylase is a Ca^2+^-dependent metal enzyme (Fig. 1a) in *Clostridium* sp. strain BOH3. The transcription levels of involved *amyA* and *amyB* genes (encoding α-amylase) and activity assays in vitro for corresponding amylolytic enzyme reveal that the CaCO_3_-mediated increase in the level of cassava saccharification stems from the enhanced α-amylase activity. In comparison, the pre-hydrolysis of starch substrate either by commercial enzymes or by acids at high temperature has negative effects, leading to costly and rigorous handling procedures. Furthermore, the treatment of substrate by acids results in a low sugar yield and the formation of harmful by-products, including formate, furfurol, and melanoids that can seriously inhibit the growth of the butanol producing *Clostridium* [29–31]. Another attempt to increase substrate utilization and butanol yield has been made to use a coculture of *Clostridium* and other organisms in which the substrate was first hydrolyzed by a fungus/*Clostridium* mixture to produce amylase, and then butanol production was achieved separately by adding another *Clostridium* species [32]. However, cocultures are subjected to carbon- and energy-intensive process and hard to maintain redox balance of fermentative bacteria cells to facilitate the more metabolite butanol production in these systems. Whether these strategies work economically still remains an open question and depends on whether the drawbacks of the respective technologies can be overcome by new developments [33]. In this case, the dual contribution (metal cofactor and buffer capacity) of CaCO_3_ was demonstrated to be the critical factor for improving cassava saccharification and ABE fermentation without pretreatment.

The availability of more reducing cofactor can direct the carbon flow from acetone and acid to alcohol production (Figs. 5, 6). This is because butanol and ethanol formation requires the reducing equivalents in the form of NADH and NADPH, whereas acid and acetone production does not (Fig. 4). Various approaches have been applied to increase the availability of NADH for butanol biosynthesis by applying redox engineering of genetically modifying the organism to rebalance the redox [34] or “Metabolic Process Engineering” (MPE) that manipulates flux distributions in the metabolic pathway through the use of artificial electron carriers such as methyl viologen [35]. In this current study, reducing cofactors were regulated by de novo synthesis pathway with the addition of precursor l-tryptophan, which increased the level of NADH and NADPH and possibly complemented with the lack of reducing cofactors in the alcohol-dependent pathways of strain BOH3. Consequently, under the defined conditions, a significant shift in the metabolic directions towards the production of more reduced metabolites (e.g., 17.8 g/L butanol) occurred, as evidenced by improved butanol–acetone ratio (76 %) and butanol–bioacid ratio (500 %) (no accumulation of acetic acid) in the reactor fermentation (Fig. 6). In contrast, early attempts to redirect metabolite flux have been made to overexpress *aad* (encoding alcohol/aldehyde dehydrogenase, AAD) gene so as to improve the butanol selective production in a *C. acetobutylicum* mutant M5 [36]. However, large amounts of acetate (14.9 g/L) and butyrate (7.7 g/L) were accumulated due to the inability of the mutant M5 to re-assimilate acid products, and butanol concentration never exceeded the amount (11.1 g/L) as generated by a wild-type *C. acetobutylicum*. In this study, adjusting the levels of NADH and NADPH by salvaging pathway from *de novo* synthesis (precursor l-tryptophan) could be an efficient approach in improving butanol production, and enough NADH and NADPH and/ or higher NADH/NAD^+^ and NADPH/NADP^+^ ratios benefit the cells in accelerating the butanol production so as to change the final distribution of the metabolites. On the other hand, manipulating cofactors may also provide an alternative means to determine cellular metabolism, in particular the interplay between cofactor levels and metabolic fluxes [37].

## Conclusions

Using the metabolism of cassava-based butanol production in *Clostridium* sp. strain BOH3 as a model system, we demonstrated that manipulation of metal and reducing cofactor levels could be used as a novel tool to improve saccharification and fermentation efficacy and to redistribute the carbon flux in the cofactor-dependent production system without pretreatment. Calcium carbonate enhanced substrate hydrolysis capacity, alcohol productivity with acetone reduction due to both its buffering effects and its role to the direct enhancement of the activities of key hydrolytic and solventogenic enzymes. The carbon flux to ethanol and butanol was shut down when reducing cofactor equivalents in the form of NADH and NADPH were lacking at the node of alcohol-dependent pathways. Interestingly, NADH and NADPH de novo synthesis in the presence of l-tryptophan complemented the availability and levels of reducing cofactor-dependent alcohol production and opened the valve of the carbon flux selectively to synthesize butanol. While molecular technologies focusing on strain construction are becoming attractive in the development of economically viable biobutanol production, metabolic technologies should not be overlooked because they can be effective means of fully exploiting the productivity of a strain and maximizing the production efficiency.

## Methods

### Organism and culture conditions

A wild-type *Clostridium* sp. strain BOH3, isolated in a previous study [38], was used throughout this study. Medium composition for seed culture included 30 g/L cassava flour, 0.2 g/L KH_2_PO_4_, 0.3 g/L NH_4_Cl, 0.5 g/L MgCl_2_·6H_2_O, 0.3 g/L KCl, 1.0 g/L NaCl. The fermentation medium consisted of cassava flour (100 g/L), mineral salts (1.0 g/L KH_2_PO_4_, 0.2 g/L MgSO_4_, 0.05 g/L MnSO_4_, 0.01 g/L FeSO_4_·7H_2_O, and 1.0 g/L NaCl), 2.2 g/L CH_3_COONH_4_, plus trace element solution (0.006 mg/L H_3_BO_3_, 0.024 mg/L NiCl_2_·6H_2_O, 0.1 mg ZnCl_2_, 1.9 mg/L CoCl_2_·6H_2_O, 0.036 mg/L Na_2_MoO_4_·2H_2_O, and 0.05 mg/L CuCl_2_·2H_2_O). l-Tryptophan was prepared as a sterile stock solution and added as necessary to the medium before fermentation. CaCO_3_ was sterilized by dry heat sterilization at 160 °C for 30 min before being added into the medium. Anaerobic medium (pH 6.0) was prepared with an addition of 0.0242 g/L l-cysteine and 0.048 g/L Na_2_S·6H_2_O, respectively [39]. Cassava flour was purchased from a supermarket (Fairprice, Singapore), and other chemicals in the study were of at least analytical-grade purity and were purchased from Sigma-Aldrich, USA.

### Experimental procedures

To determine the dose-dependent effects of CaCO_3_ on cassava hydrolysis and ABE fermentation by a wild-type *Clostridium* species strain BOH3, fermentations (in 160 mL serum bottles containing 47 mL fermentation medium plus 3 mL active inocula (6 %, v/v) with an OD of ~3) were conducted for 120 h in a defined medium supplemented with 5, 10, 30, 50, 70 or 100 mM of CaCO_3_. The medium without CaCO_3_ was used as a negative control. Unless otherwise stated, all fermentation experiments were conducted in triplicates, and the cultures were incubated at 37 °C and shaking at 150 rpm without pH control. Samples were taken periodically for analysis of starch hydrolysis and ABE production.

We then evaluated the role of CaCO_3_ on cassava hydrolysis and butanol production. Four experiments were designed in serum bottles to test whether Ca^2+^ or CO_3_^2‒^ or both of them stimulated improvements of ABE fermentation: (1) absence of CaCO_3_ (negative controls as blank); (2) supplementing with 10 mM CaCl_2_ (for investigating the effects of Ca^2+^ ion on α-amylase activity and butanol formation); (3) supplementing with 10 mM Na_2_CO_3_ (for investigating the effects of CO_3_^2−^ ion on α-amylase activity and butanol formation); (4) supplementing with those of (2) plus (3) being added simultaneously (for investigating effects of both Ca^2+^ and CO_3_^2−^ ions on α-amylase activity and butanol production); (5) supplementing with 10 mM CaCO_3_ (the positive control). All culture media were inoculated with 6 % (v/v) active BOH3 cells (OD of ~3) and the cultures were incubated for 120 h in an incubator shaker as described above. All experiments were conducted in triplicates without pH control.

To further assess the potency of l-tryptophan in enhancing availability and reducing potential of NADH and NADPH, and activity of butanol dehydrogenase in culture BOH3, various amount of l-tryptophan (0.5–2.5 g/L) was dosed to the cassava-defined medium. Medium without l-tryptophan was used as the negative control.

To integrate butanol production and cassava hydrolysis, bioreactor fermentations were carried out in a 3 L stirred-tank bioreactor (BIOSTAT^®^ B plus, Sartorius, Germany) at 37 °C and at 150 rpm. The oxygen entrapped in the bioreactor (in the headspace of the bioreactor and in the bulk liquid) was removed by sparging with pure nitrogen through a 0.2-μm filter for 40 min before sterilization. Actively growing BOH3 cells (OD of ~3) of 90 ml were inoculated into the bioreactor containing 1.5 L medium. The initial pH of the fermentation was 6.0, and the pH was allowed to drop to 5.0 as the culture progressed. Subsequently, the pH was automatically maintained at or above 5.0 by adding 9 M ammonium hydroxide [17]. All experiments were conducted in triplicate. Samples were collected at various time intervals and centrifuged at 10,000×*g* for 15 min at 4 °C to determine cell growth, metabolites, and residual starch concentration. The enzyme activity assays, transcriptional expression levels of functional genes involved in starch hydrolysis and solventogenesis, and intracellular cofactor measurements (Ca^2+^) were also implemented after the inoculation.

### Analytical methods

Cell concentration was measured using a spectrophotometer (Biospec-1601; Shimadzu Co., Kyoto, Japan) at 600 nm after proper dilution. The optical density (OD_600_) was converted to dry cell weight (DCW) according to a predetermined calibration line (OD_600_:DCW (g/L) = 1:0.36). The concentrations of the fermentation metabolites including acetone, butanol, ethanol, acetate, and butyrate were measured by gas chromatography (Agilent 7890A, Agilent, USA) equipped with a J & W 123-7334 column (30 m × 320 μm × 0.25 μm) and a flame ionization detector, as described previously [17]. Residual glucose concentrations were quantified with a biochemical analyzer (YSI 2700, USA). Cassava starch was assayed by a starch assay kit according to the manufacturer’s protocol (A4582, Sigma-Aldrich, USA). Cassava flour used in this study contained 68 % starch (w/w) and the rest components (e.g., ash, moisture, fiber, nitrogen, and inorganics, etc.) are considered as non-fermentable.

### Assay of extracellular hydrolytic α-amylase

The assay for α-amylase activity involved measurement of the reducing sugar from the enzymatic hydrolysis of soluble starch. The reaction mixture consisted of 1.25 mL of 2 % soluble starch, 0.5 mL of 0.2 M acetate buffer (pH 5.0), and a 0.25 mL supernatant sample. After 10 min of incubation at (30 °C), the reaction was stopped by boiling at 100 °C for 10 min. The control was carried out in the same manner using a sample previously inactivated by boiling for 15 min. The liberated reducing sugars were estimated by the dinitrosalicylic acid (DNS) method with glucose as the standard. One unit (U) of α-amylase activity was defined as the amount of enzyme that released 1 µmol of glucose equivalent per min under the defined conditions.

### Assay of intracellular enzyme

Crude cell extracts of culture BOH3 were assayed for the involved solventogenic enzymes (BADH, BDH, AAD, and AADC). Anaerobic conditions were maintained throughout the entire procedure. Ten milliliter of cells was harvested by centrifugation at 10,000×*g* for 5 min at 4 °C. The cell pellets were re-suspended in ice-cold respective buffers (35 mM Tris–HCl, pH 7.5) for BADH and BDH, and (50 mM sodium acetate, pH 5) for AADC. Cell lysis was achieved by ultrasonication on ice for 15 min using a 20 kHz ultrasonicator (VCX 130, Sonics & Materials Inc., CT, USA) with the following conditions: 5 s of sonication with a 10-s interval, set at 50 % amplitude. The resulting lysate was then collected and centrifuged at 14,000 rpm at 4 °C for 20 min to remove cell debris. The supernatant was then retrieved for subsequent enzyme assays. Protein concentration of cell extracts was determined using the DC protein assay Kit (BioRad, USA). Unless stated otherwise, all enzyme assays were performed in an anaerobic workstation at 30 °C with 10 × concentrated cell extracts. NADH- and NADPH-dependent BADH or BDH activities were assayed at 340 nm, as described previously [40, 41]. One unit was defined as the amount of enzyme required to oxidize 1 µmol of NADH or NADPH per min with butyryl-CoA or butyraldehyde as the substrate. AADC activity was determined as described previously [42]. One unit of AADC activity was defined as the amount of CO_2_ (in microliters) produced per minute per mg protein. All values of enzymatic assay were averaged values of at least three independent extract procedures.

### Determination of Ca^2+^ levels in α-amylase and in cell extract of culture BOH3

Purification of α-amylase of *Clostridium* sp. strain BOH3 was performed by fast protein liquid chromatography (FPLC) (AKTA purifier, GE Healthcare, USA). Briefly, HiTrap ANX (diethylaminopropyl) Sepharose fast-flow weak anionic exchange column with a volume of 1 mL was used. The supernatant containing crude enzymes was adjusted to pH 7.4 by adding 1 M of NaOH. Subsequently, it was filtered through a 0.22 μm membrane filter before loading into the ANX column. The binding buffer was Tris buffer (50 mM, pH 7.2) and elution buffer was Tris buffer with 1 mol/L of NaCl. Both binding and elution buffers were filtered through a 0.22 μm membrane filter (Sartorius, USA) using a vacuum filtration unit. The elution was conducted using step gradient (25, 50, 75, and 100 %) method with a flow rate of 1 mL/min in FPLC, and 5 mL fractions were collected during the elution process.

Intracellular Ca^2+^ concentration was quantified by a fluorescent Calcium Green-1 Ca^2+^ indicator kit (C3010MP, Life Technologies, USA) according to the manufacturer’s protocol. Briefly, stock solutions (10 mM) of fluorescent Ca^2+^ indicator were made using a physiological buffer (100 mM KCl and 10 mM MOPS, pH 7.2). Aliquots were diluted with the buffer to give a final concentration of 10 µM. The resulting supernatants of cell extracts and purified α-amylase elutes were loaded and labeled with the fluorescent Ca^2+^ indicator at room temperature in the dark for 20 min. Intracellular Ca^2+^ concentration was determined in a plate-based fluorometer (Infinite 200 pro, Tecan, Switzerland) with the excitation/emission maxima of 506 and 531 nm.

### NAD^+^/NADH and NADP^+^/NADPH assay

NAD^**+**^/NADH or NADP^**+**^/NADPH levels were measured with Amplite™ Fluorimetric NAD/NADH or NADP/NADPH ratio assay kits (ATT Bioquest, CA) as previously described [17]. Briefly, cells were harvested by centrifugation at 14,000 rpm at 4 °C. The pellets were then re-suspended with 0.2 mL PBS buffer (pH 7.4) and 0.2 mL NAD^**+**^/NADH or NADP^**+**^/NADPH lysis buffer provided in the assay kits. Lysis was allowed to proceed for 15 min at room temperature until the cell re-suspension turned clear. The lysate was then centrifuged at 1500 rpm for 5 min at 4 °C. The supernatant was retrieved for subsequent NAD^**+**^/NADH or NADP^**+**^/NADPH assays. For the measurement of intracellular NAD^**+**^/NADH or NADP^**+**^/NADPH levels, 25 μL of cell lysates was treated with or without NADH/NAD^**+**^ or NADPH/NADP^**+**^ extraction solution for 15 min, and then neutralized with extraction solutions at room temperature, and incubated the reaction at room temperature in the dark for 30 min after adding 75 μL of NADH or NADPH reaction mixture. The readings were taken by running a 96-well black plate on a fluorescence microplate reader (Infinite 200 pro, Tecan, Switzerland) at Ex/Em = 530–570/590–600 nm (maximum Ex/Em = 540/590 nm). The blank signal was subtracted from the values for those wells with the NADH or NADPH reactions.

### RNA isolation and reverse transcription-PCR analysis

*Clostridium* sp. strain BOH3 cells were incubated in the presence and the absence (control) of CaCO_3_. At each time point, 1 mL of culture was harvested for RNA or DNA extraction, by pelleting at 10,000*g*, 4 °C for 10 min (all duplicate). DNA extraction was done using QIAGEN DNeasy Blood and Tissue kit (QIAGEN GmbH, Hilden, Germany), which was used for enumerization of cell numbers in the culture by quantitative real-time PCR (qPCR). Pellets for RNA extraction were immediately re-suspended in Trizol reagent (Invitrogen) and stored at −80 °C. Before extraction, 2 µL of Luciferase mRNA control (Promega) (diluted to 10^8^ copies/µL) was added into each sample to quantify the loss during extraction and reverse transcription. Supernatant after chloroform treatment was mixed with an equal volume of 70 % (v/v) chilled ethanol before loading onto the QIAGEN RNeasy mini column. Possible residual genomic DNA was removed by on-column incubation with QIAGEN RNase-Free DNase Set. RNA was eluted using RNase-free water. Reverse transcription (RT) was performed immediately after RNA elution using ABI High Capacity cDNA RT kit with random hexamer primer (Promega) in the presence of RNasin RNase inhibitor (Promega). qPCR was performed in 20-µL reactions using Bioline SensiFAST SYBR Lo-ROX Kit on an ABI 7500 FAST Real-Time PCR system. Primers used in this study are listed in Additional file 1: Table S1. The RT-PCR conditions were as follows: 2 min at 95 °C and then 45 cycles of 5 s at 95 °C and 30 s at 60 °C, followed by heating from 55 to 95 °C with a ramp speed of 1 °C per 10 s. Luciferase cDNA copies were quantified using primers LucF and LucR. Transcripts of individual genes were quantified using primers specifically designed based on their gene sequences. Numbers of gene transcripts per cell were obtained by normalizing transcript copies based on luciferase losses, and then divided by cell numbers at each time point.

### Nucleotide sequence accession numbers

The functional gene sequences of *amyA*, *amyB*, *bdhA*, *bdhB*, *bdhC*, *bdhD*, *aadA*, *aadB*, and *adc* of *Clostridium* sp. strain BOH3 have been deposited in GenBank under accession numbers KT362051 to KT362059, respectively.

## Additional file

10.1186/s13068-015-0351-7 Oligonucleotide primers used in the RT-PCR.

## Abbreviations

ABE: acetone–butanol–ethanol
SSF: simultaneous saccharification and fermentation
NADH: nicotinamide adenine dinucleotide hydrogen
NADPH: nicotinamide adenine dinucleotide phosphate hydrogen
NAD^+^: nicotinamide adenine dinucleotide
NADP^+^: nicotinamide adenine dinucleotide phosphate
α-AMY: α-amylase
BADH: butyraldehyde dehydrogenase
BDHA: butanol dehydrogenase I
BDHB: butanol dehydrogenase II
BDHC: butanol dehydrogenase III
AAD: alcohol/aldehyde dehydrogenase
AADC: acetoacetate decarboxylase
RT: reverse transcription
PCR: polymerase chain reaction
DCW: dry cell weight
OD: optical density

## References

[1] Green EM (2011). Fermentative production of butanol—the industrial perspective. Curr Opin Biotechnol.

[2] Kumar M, Gayen K (2011). Developments in biobutanol production: new insights. Appl Energy.

[3] Lee SY, Park JH, Jang SH, Nielsen LK, Kim J, Jung KS (2008). Fermentative butanol production by clostridia. Biotechnol Bioeng.

[4] Chiao JS, Sun ZH (2007). History of the acetone–butanol–ethanol fermentation industry in China: development of continuous production technology. J Mol Microbiol Biotechnol.

[5] Liu D, Chen Y, Ding FY, Zhao T, Wu JL, Guo T, Ren HF, Li BB, Niu HQ, Cao Z, Lin XQ, Xie JJ, He XJ, Ying HJ (2014). Biobutanol production in a *Clostridium acetobutylicum* biofilm reactor integrated with simultaneous product recovery by adsorption. Biotechnol Biofuels.

[6] Ezeji TC, Qureshi N, Blaschek HP (2004). Butanol fermentation research: upstream and downstream manipulations. Chem Rec.

[7] Tran HTM, Cheirsilp B, Hodgson B, Umsakul K (2010). Potential use of *Bacillus subtilis* in a co-culture with *Clostridium butylicum* for acetone–butanol–ethanol production from cassava starch. Biochem Eng J.

[8] FAO. Food Outlook: Global Market Analysis. Food and Agriculture Organisation. 2012. http://www.fao.org/docrep/016/al993e/al993e00.pdf. Accessed 2 Nov 2012.

[9] Thang VH, Kobayashi G (2014). A novel process for direct production of acetone–butanol–ethanol from native starches using granular starch hydrolyzing enzyme by *Clostridium saccharoperbutylacetonicum* N1-4. Appl Biochem Biotechnol.

[10] Ezeji TC, Qureshi N, Karcher P, Blaschek HP, Minteer S (2006). Production of butanol from corn. Alcoholic Fuels.

[11] Thang VH, Kanda K, Kobayashi G (2010). Production of acetone–butanol–ethanol (ABE) in direct fermentation of cassava by *Clostridium saccharoperbutylacetonicum* N1-4. Appl Biochem Biotechnol.

[12] Soni BK, Kapp C, Goma G, Soucaille P (1992). Solvent production from starch: effect of pH on α-amylase and glucoamylase localization and synthesis in synthetic medium. Appl Microbiol Biotechnol.

[13] Trovati J, Giordano RC, Giordano RLC (2009). Improving the performance of a continuous process for the production of ethanol from starch. Appl Biochem Biotechnol.

[14] Gupta R, Gigras P, Mohapatra H, Goswami VK, Chauhan B (2003). Microbial α-amylases: a biotechnological perspective. Process Biochem.

[15] Malhotra R, Noorvez SM, Satyanarayana T (2000). Production and partial characterization of thermostable and calcium independent alpha amylase of an extreme thermophile *Bacillus thermoleovorans* NP54. Lett Appl Microbiol.

[16] Sivaramakrishnan S, Gangadharan D, Nampoothiri KM, Soccol CR, Pandey A (2006). α-Amylases from microbial sources. Food Technol Biotechnol.

[17] Li TG, Yan Y, He J (2014). Reducing cofactors contribute to the increase of butanol production by a wild-type *Clostridium* sp. strain BOH3. Bioresour Technol.

[18] Berríos-Rivera SJ, Bennett GN, San KY (2002). The effect of increasing NADH availability on the redistribution of metabolic fluxes in *Escherichia coli* chemostat cultures. Metab Eng.

[19] Knepper A, Schleicher M, Klauke M, Weuster-Botz D (2008). Enhancement of the NAD(P)(H) pool in *Saccharomyces cerevisiae*. Eng Life Sci.

[20] Burhan A, Nisa U, Gokhan C, Omer C, Ashabil A, Osman G (2003). Enzymatic properties of a novel thermostable, thermophilic, alkaline and chelator resistant amylase from an alkaliphilic *Bacillus* sp. isolate ANT-6. Process Biochem.

[21] Chung YC, Kobayashi T, Kanai H, Akiba T, Kudo T (1995). Purification and properties of extracellular amylase from the hyperthermophilic archeon *Thermococcus profundus* DT5432. Appl Environ Microbiol.

[22] Zheng Y, Bruice T (1997). Conformation of coenzyme pyrroloquinoline quinone and role of Ca^2+^ in the catalytic mechanism of quinoprotein methanol dehydrogenase. Proc Natl Acad Sci USA.

[23] Vangnai AS, Arp DJ (2001). An inducible 1-butanol dehydrogenase, a quinohaemoprotein, is involved in the oxidation of butane by ‘*Pseudomonas butanovora*’. Microbiology.

[24] Li L, Ai HX, Zhang SX, Li S, Liang ZX, Wu ZQ, Yang ST, Wang JF (2013). Enhanced butanol production by coculture of *Clostridium beijerinckii* and *Clostridium tyrobutyricum*. Bioresour Technol.

[25] Li HG, Qiang WL, Yu XB (2014). Direct fermentation of gelatinized cassava starch to acetone, butanol, and ethanol using *Clostridium acetobutylicum* mutant obtained by atmospheric and room temperature plasma. Appl Biochem Biotechnol.

[26] Virunanon C, Ouephanit C, Burapatana V, Chulalaksananukul W (2013). Cassava pulp enzymatic hydrolysis process as a preliminary step in bio-alcohols production from waste starchy resources. J Clean Prod.

[27] Jiang Y, Xu CM, Dong F, Yang YL, Jiang WH, Yang S (2009). Disruption of the acetoacetate decarboxylase gene in solvent-producing *Clostridium acetobutylicum* increases the butanol ratio. Metab Eng.

[28] Han B, Ujor V, Lai LB, Gopalan V, Ezeji TC (2013). Use of proteomic analysis to elucidate the role of calcium in acetone–butanol–ethanol fermentation by *Clostridium beijerinckii* NCIMB 8052. Appl Environ Microbiol.

[29] Wang SH, Zhang YP, Dong HJ, Mao SM, Zhu Y, Wang RJ, Luan GD, Li Y (2011). Formic acid triggers the “acid crash” of acetone–butanol–ethanol fermentation by *Clostridium acetobutylicum*. Appl Environ Microbiol.

[30] Zhang Y, Ezeji TC (2013). Transcriptional analysis of *Clostridium beijerinckii* NCIMB 8052 to elucidate role of furfural stress during acetone butanol ethanol fermentation. Biotechnol Biofuels.

[31] Ezeji TC, Qureshi N, Blaschek HP (2007). Butanol production from agricultural residues: impact of degradation products on *Clostridium beijerinckii* growth and butanol fermentation. Biotechnol Bioeng.

[32] Weber C, Farwick A, Benisch F, Brat D, Dietz H, Subtil T, Boles E (2010). Trends and challenges in the microbial production of lignocellulosic bioalcohol fuels. Appl Microbiol Biotechnol.

[33] Wingren A, Galbe M, Zacchi G (2003). Techno-economic evaluation of producing ethanol from softwood: comparison of SSF and SHF and identification of bottlenecks. Biotechnol Prog.

[34] Shen CR, Lan EI, Dekishima Y, Baez A, Cho KM, Liao JC (2011). Driving forces enable high-titer anaerobic 1-butanol synthesis in *Escherichia coli*. Appl Environ Microbiol.

[35] Du YM, Jiang WY, Yu MR, Tang I-C, Yang S-T (2015). Metabolic process engineering of *Clostridium tyrobutyricum* Δ*ack*-*adhE2* for enhanced n-butanol production from glucose: effects of methyl viologen on NADH availability, flux distribution, and fermentation kinetics. Biotechnol Bioeng.

[36] Sillers R, Chow A, Tracy B, Papoutsakis ET (2008). Metabolic engineering of the non-sporulating, non-solventogenic *Clostridium acetobutylicum* strain M5 to produce butanol without acetone demonstrate the robustness of the acid-formation pathways and the importance of the electron balance. Metab Eng.

[37] Li ZG, Shi ZP, Xin S, Li L, Zheng JP, Wang ZG (2013). Evaluation of high butanol/acetone ratios in ABE fermentations with cassava by graph theory and NADH regeneration analysis. Biotechnol Biopro Eng.

[38] Bramono SE, Lam YS, Ong SL, He J (2011). A mesophilic *Clostridium* species that produces butanol from monosaccharides and hydrogen from polysaccharides. Bioresour Technol.

[39] He J, Ritalahti KM, Yang K-L, Koenigsberg SS, Loffler FE (2003). Detoxification of vinyl chloride to ethene coupled to growth of an anaerobic bacterium. Nature.

[40] Dürre P, Kuhn A, Gottward M, Gottschalk G (1987). Enzymatic investigations on butanol dehydrogenase and butyraldehyde dehydrogenase in extracts of *Clostridium acetobutylicum*. Appl Microbiol Biotechnol.

[41] Stim-Herndon KP, Nair R, Papoutsakis ET, Bennett GN (1996). Analysis of degenerate variants of *Clostridium acetobutylicum* ATCC 824. Anaerobe.

[42] Gerischer U, Dürre P (1990). Cloning, sequencing, and molecular analysis of the acetoacetate decarboxylase gene region from *Clostridium acetobutylicum*. J Bacteriol.

