# Supplementary material for: Enhanced direct fermentation of cassava to butanol by Clostridium species strain BOH3 in cofactor-mediated medium
Source: Biotechnol Biofuels. 2015 Oct 12;8:166. doi: 10.1186/s13068-015-0351-7 (PMC4603972; doi:10.1186/s13068-015-0351-7)
Supplement: Supplementary file 1 — 10.1186/s13068-015-0351-7 Oligonucleotide primers used in the RT-PCR. [file 13068_2015_351_MOESM1_ESM.doc]

**Enhanced direct fermentation of cassava to butanol by *Clostridium* species strain BOH3 in cofactors-mediated medium**

Tinggang Li^†^

Email: ceelt@nus.edu.sg; tglibj@hotmail.com

Yu Yan^†^

Email: a0082795@nus.edu.sg

Jianzhong He^*^

Email: jianzhong.he@nus.edu.sg

^†^These authors contributed equally to this work.

^*^Corresponding author

Department of Civil and Environmental Engineering

National University of Singapore

Block E2-02-13, 1 Engineering Drive 3

Singapore 117576

Phone: (65) 6516-3385; Fax: (65) 6779-1635

**Table S1 Oligonucleotide primers used in the RT-PCR**

| **Primer** | **Sequence (5ʹ-3ʹ)** | **Fragment size (bp)** | **Target** |
| --- | --- | --- | --- |
| amyA(F) | GGAGAATGACAGGAACAGTACCA | 105 | *amyA* |
| amyA(R) | CTTTTGCAGCTTGCTCACTTAA |
| amyB(F) | TGTAATTGCTCAAGCCTTTACCTG | 120 | *amyB* |
| amyB(R) | CCAGCCTTGGGAGTTCTTTG |
| bdhA(F) | CTGATGATTACGAGGCTAGAGCTA | 111 | *bdhA* |
| bdhA(R) | CGTGTTCCATAGGATGACAACTC |
| bdhB(F) | AGGTTATAGCAGCAGCATGTGAA | 117 | *bdhB* |
| bdhB(R) | ATCCTGTTGCAGCAATGGTTA |
| bdhC(F) | GAAGGAAGATTTCAAGACCGTACA | 165 | *bdhC* |
| bdhC(R) | ATCCTGCGGAACTCCTGCTC |
| aadA(F) | AACAAACCCCACATCAACAACA | 148 | *aadA* |
| aadA(R) | GGCACCACTCTTAACGGCTG |
| aadB(F) | GCATATGTTTCGGTTATGGCTAC | 122 | *aadB* |
| aadB(R) | TCTCTTGCTTCAATGTCGTTAGTC |
| adc(F) | TAGAGTTGCGACAGCTACAATGG | 181 | *adc* |
| adc(R) | TGTCCAAGCTTCATGTACGGTAA |
| 338q(F) | ACTCCTACGGGAGGCAGCAG | 180 | 16S *rRNA* |
| 518q(R) | ATTACCGCGGCTGCTGG |
| Luc(F) | TACAACACCCCAACATCTTCGA | 67 | Luciferase cDNA |
| Luc(R) | GGAAGTTCACCGGCGTCAT |

F, forward; R, reverse
